# Multiple ventricular septal defects: Septal reconstruction with right atrial wall

**DOI:** 10.1016/j.xjtc.2026.102425

**Published:** 2026-05-21

**Authors:** Igor E. Konstantinov, Bakhytzhan Nurkeyev, Amine Mazine, Erbol Aldabergenov, Elmira Kuandykova, Bauyrzhan Tuyakbayev, Assel Kabakanova, Arailym Kenzhebaeva, Amangeldy Kerimkulov, Zhanibek Z. Ashirov, Aidos Erpashov, Abay K. Baigenzhin

**Affiliations:** a Department of Cardiothoracic Surgery, Royal Children's Hospital, University of Melbourne, Murdoch Children's Research Institute, Melbourne Centre for Cardiovascular Genomics and Regenerative Medicine, Melbourne, Australia; b Cardiovascular Surgery, Mayo Clinic, Rochester, Minn; c National Scientific Medical Center, Astana, Kazakhstan

**Figure:**
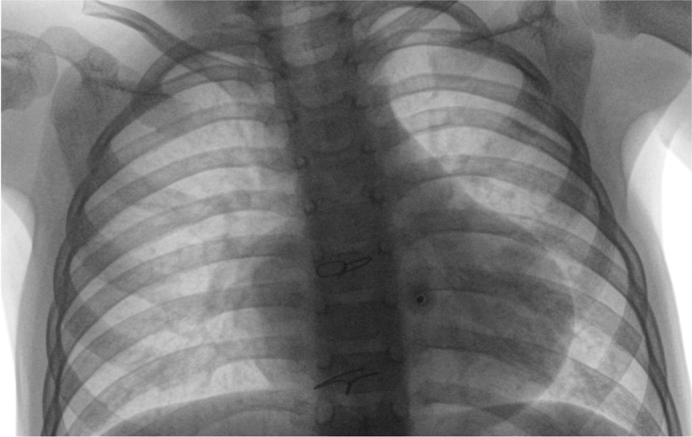
Occluder device in noncompacted ventricular septum.

**Central Message**

Ventricular septal reconstruction with right atrial wall is feasible in noncompacted ventricular septum.

Patients with multiple muscular ventricular septal defects (VSDs), often referred to as “Swiss-cheese” VSDs, frequently have a thin and poorly developed ventricular septum with features of myocardial noncompaction.^1^, ^2^, ^3^ In these patients, complete closure of all defects can be technically challenging, particularly when the septum is extensively trabeculated and dysplastic. Although precise delineation between a dysplastic ventricular septum and myocardial noncompaction is not always feasible, it is clear that many patients with “Swiss-cheese” septum exhibit features of noncompaction syndrome.^1^, ^2^, ^3^, ^4^ These patients often require complex management, including hybrid procedures as initial palliation, to achieve biventricular repair and reconstruction of the ventricular septum.^2^

We have previously described a transventricular approach to effectively close multiple VSDs in patients with “Swiss-cheese” septum. An ideal patch material for septal reconstruction should consist of autologous living tissue and remain sufficiently compliant to avoid stiffening of the septum and preserve both systolic and diastolic biventricular function.

Herein, we describe the use of autologous right atrial (RA) appendage for the reconstruction of a severely dysplastic ventricular septum with multiple VSDs in a patient with “Swiss-cheese” septum and features of noncompaction. This approach allows complete septal reconstruction using a single patch under direct visualization.

## Operative Technique

All procedures described in this report were performed in the Children's Hospital, National Scientific Medical Center in Astana, Kazakhstan. This retrospective study was approved by the hospital research ethics committee (HREC 07-2025; July 1, 2025), and informed consent for publication was obtained from the parents.

A 2.5-year-old girl (weight 12 kg; height 94 cm; body surface area 0.47 m^2^) with known multiple VSDs presented with exercise intolerance. She had undergone patent ductus arteriosus ligation and pulmonary artery banding at 5 months of age and hybrid closure of the largest muscular VSD with occluder at the age of 1 year. The initial palliation was performed because of ventricular dilatation and decreased biventricular function with characteristics of noncompacted myocardium.

**Figure 1:**
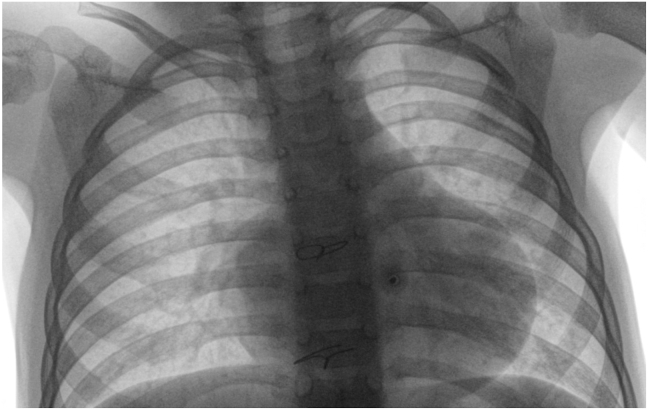
Radiographic image after occluder device placement and pulmonary artery banding in a child with multiple ventricular septal defects.

**Figure 2:**
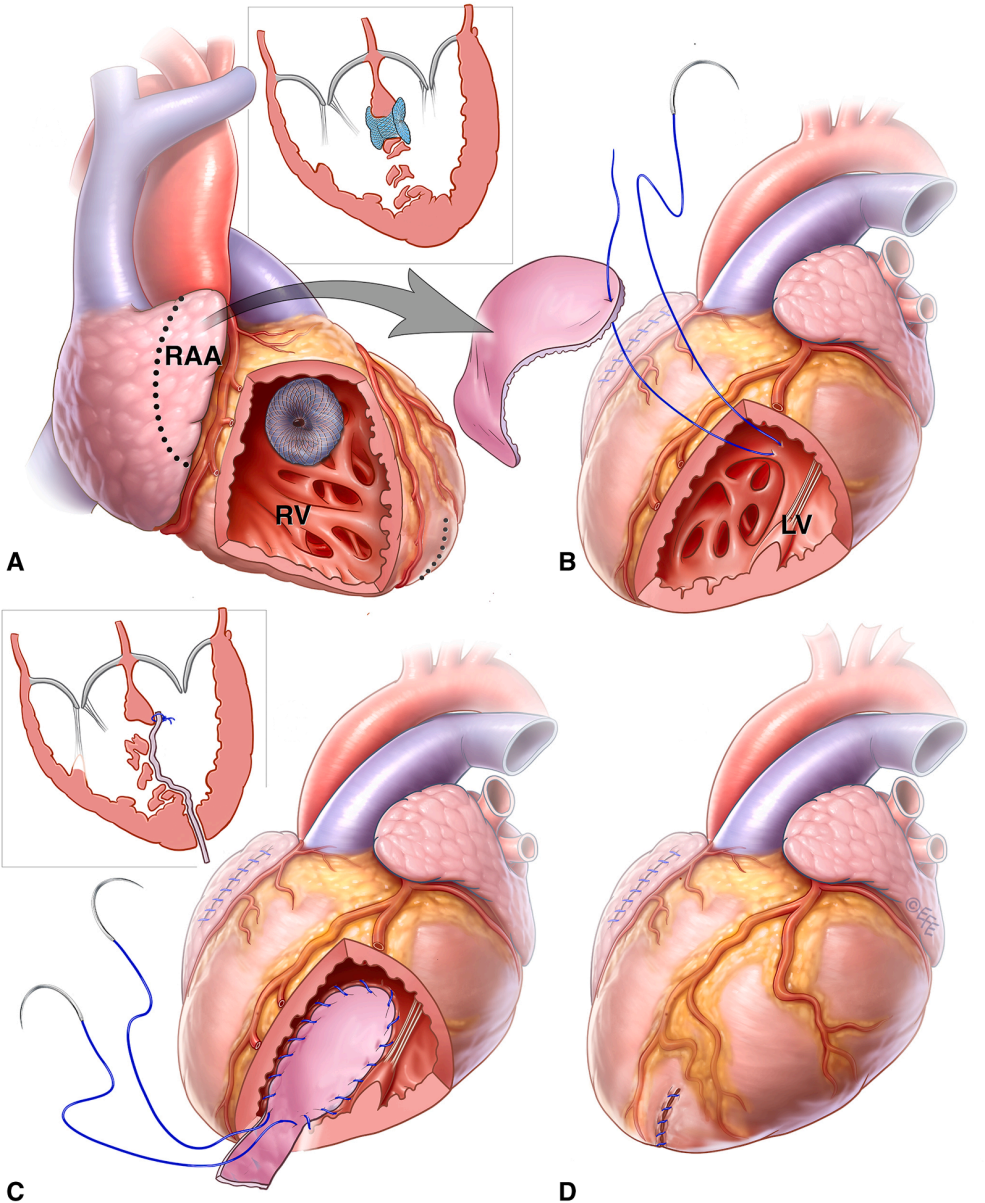
Technique of septal reconstruction with right atrial wall patch in a patient with noncompacted ventricular septum and multiple ventricular septal defects. A and B, The trabeculated part of the atrial septum is taken to be used as a patch for ventricular septum reconstruction. C and D, The apex of the LV is opened, and the right atrial patch is placed to restore the ventricular septum with the smooth epicardial surface of the patch facing the LV cavity. The ventriculotomy is closed, incorporating the edge of the right atrial patch into the suture line. *RAA*, right atrial appendage; *LV*, left ventricle/ventricular; *RV*, right ventricle.

At the time of her current presentation (Figure 1, Video 1) she had preserved left ventricular dimensions and function (left ventricular ejection fraction 59%, left ventricular end-diastolic diameter 3.1 cm). The peak gradient across the pulmonary artery band was 65 mm Hg and the mean was 35 mm Hg. Echocardiography demonstrated a very thin ventricular septum, with multiple VSDs and VSD occluder that appeared to make the proximal muscular septum immobile. After sternotomy, a cardiopulmonary bypass with bicaval cannulation was instituted, the heart was arrested, and an incision in the left ventricular apex was made (Figure 2, Video 1). The appendage of the RA was harvested and used as a patch to close multiple VSDs and reconstruct dysplastic ventricular septum. Cardiopulmonary bypass time was 138 minutes. Aortic crossclamp time was 89 minutes. The patient had an uneventful recovery. She is doing very well and is asymptomatic with normal exercise tolerance at 12 months after surgery. Echocardiographic assessment confirmed normal biventricular function and intact ventricular septum.

## Discussion

Closure of the multiple VSDs can be problematic, especially in patients with decreased function, and may require preoperative palliative procedures.^5^^,^^E1^ In patients with a severely dysplastic or noncompacted ventricular septum, exposure from the right-sided approach may not permit reliable identification and closure of all defects. Re-endocardialization may result in plication of the septum and has been associated with impaired diastolic function; in addition, it may not allow complete closure in this anatomy.

Although the long-term outcomes of ventricular septal reconstruction using a living autologous RA patch remain to be determined, the softness and thickness of the trabeculated RA appendage appear to provide an ideal combination. The tissue is sufficiently soft and compliant to avoid interference with ventricular contraction while allowing normal relaxation and filling. The smooth epicardial surface may theoretically minimize thrombogenesis in patients with reduced left ventricular function, whereas atrial wall trabeculations appear to integrate well within the myocardial mesh of the dysplastic, multiperforated septum. Because the patch is placed from the left ventricular site, it is pressed into the septal trabecular mesh by greater left ventricular pressure. The native mesh of ventricular septal trabeculations supports the patch and may reduce the risk of dehiscence or aneurysmal dilatation. In addition, a left apical ventriculotomy provides excellent visualization of the entire ventricular septum. Previous reports suggest that apical ventriculotomy does not appear to adversely affect ventricular function after the repair of multiple muscular VSDs, although long-term data remain limited.^E2^ Furthermore, the use of autologous right atrial wall as reconstructive tissue has also been described in other congenital cardiac repairs, including augmentation of dysplastic tricuspid valve leaflets, where it provides a compliant living autologous patch with favorable handling characteristics and early functional results.^E3^ In summary, although long-term results remain to be established, ventricular septal reconstruction using living autologous right atrial wall is a simple and reproducible technique that provides excellent early results.

## Conflict of Interest Statement

The authors reported no conflicts of interest.

The *Journal* policy requires editors and reviewers to disclose conflicts of interest and to decline handling or reviewing manuscripts for which they may have a conflict of interest. The editors and reviewers of this article have no conflicts of interest.

## Supplementary Data

Video 1 The senior author explains perioperative findings and procedures. Video available at: https://www.jtcvs.org/article/S2666-2507(26)00232-4/fulltext.

## References

[1] Lopez L., Houyel L., Colan S.D. (2018). Classification of ventricular septal defects for the eleventh iteration of the international classification of diseases-striving for consensus: a report from the International Society for Nomenclature of Paediatric and Congenital Heart Disease. Ann Thorac Surg.

[2] AlFadley F., Abdelbaky N., Alhabdan M. (2022). Association of myocardial muscle non-compaction and multiple ventricular septal defects by echocardiography. Pediatr Cardiol.

[3] Moorthy N., Jain S., Neyaz Z., Kumar S., Goel P.K. (2015). Left ventricular non-compaction with multiple ventricular septal defects. Heart Views.

[4] Konstantinov I.E., Schulz A., Buratto E. (2022). Trans-ventricular approach to “Swiss-cheese” ventricular septal defects: septal exclusion technique. Op Tech Thorac Cardiovasc Surg.

[5] Daley M., Brizard C.P., Konstantinov I.E. (2019). Outcomes of patients undergoing surgical management of multiple ventricular septal defects. Semin Thorac Cardiovasc Surg.

